# Early Diagnosis of Tularemia by Flow Cytometry, Czech Republic, 2003–2015^1^

**DOI:** 10.3201/eid2510.181875

**Published:** 2019-10

**Authors:** Aleš Chrdle, Pavlína Tinavská, Olga Dvořáčková, Pavlína Filipová, Věra Hnetilová, Pavel Žampach, Květoslava Batistová, Václav Chmelík, Amanda E. Semper, Nick J. Beeching

**Affiliations:** České Budějovice Hospital, České Budějovice, Czech Republic (A. Chrdle, P. Tinavská, P. Filipová, V. Hnetilová, P. Žampach, V. Chmelík);; University of South Bohemia Faculty of Health and Social Sciences, České Budějovice, Czech Republic (A. Chrdle, O. Dvořáčková);; Royal Liverpool University Hospital, Liverpool, UK (A. Chrdle, N.J. Beeching);; Písek Hospital, Písek, Czech Republic (K. Batistová);; Public Health England, Porton Down, UK (A.E. Semper);; National Institute for Health Research Health Protection Research Unit in Emerging and Zoonotic Infections, Liverpool, UK (A.E. Semper; N.J. Beeching);; Liverpool School of Tropical Medicine, Liverpool (N.J. Beeching)

**Keywords:** tularemia, *Francisella tularensis*, T cells, flow cytometry, diagnosis, gamma delta T cells, γδ T cells, double-negative T cells, Czech Republic, early diagnosis, bacteria, peripheral blood, ROC curve, intracellular pathogens, work-up

## Abstract

We retrospectively assessed the utility of a flow cytometry–based test quantifying the percentage of CD3+ T cells with the CD4–/CD8– phenotype for predicting tularemia diagnoses in 64 probable and confirmed tularemia patients treated during 2003–2015 and 342 controls with tularemia-like illnesses treated during 2012–2015 in the Czech Republic. The median percentage of CD3+/CD4–/CD8– T cells in peripheral blood was higher in tularemia patients (19%, 95% CI 17%–22%) than in controls (3%, 95% CI 2%–3%). When we used 8% as the cutoff, this test’s sensitivity was 0.953 and specificity 0.895 for distinguishing cases from controls. The CD3+/CD4–/CD8– T cells increased a median of 7 days before tularemia serologic test results became positive. This test supports early presumptive diagnosis of tularemia for clinically suspected cases 7–14 days before diagnosis can be confirmed by serologic testing in regions with low prevalences of tularemia-like illnesses.

Tularemia is a zoonotic disease that occurs in the Northern Hemisphere and is caused by *Francisella tularensis* (*1*). In Europe, >500 cases are reported annually (*2*); Turkey and the United States also have substantial disease burdens (*3*). The infection is usually acquired by direct contact with, inhalation of, or ingestion of *F. tularensis* from animal reservoirs, infected arthropod vectors, or contaminated water (*4*).

No clinical or laboratory manifestations are pathognomonic for tularemia; preliminary diagnosis is based on exposure risk and compatible clinical presentation (*5*). Clinical manifestations include the ulceroglandular, glandular, oroglandular, and oculoglandular forms; septicemic (typhoidal) form; and respiratory form (*6*,*7*).

The decision to treat patients for tularemia is often based on clinical judgment, and therapy is initiated empirically days to weeks before the diagnosis is confirmed because seroconversion can take 10–20 days after symptom onset to occur (*8*). Culturing *F. tularensis* requires special handling because the bacterium is fastidious and needs to be cultured in Biosafety Level 3 facilities (*9*). To perform nucleic acid amplification testing, a sample from a swab of an ulcer or biopsy of deep tissue is required. A diagnostic test that is less invasive than tissue biopsy and supports early diagnosis of tularemia would be beneficial in guiding empiric therapy.

Peripheral blood CD3+ T lymphocytes that do not exhibit CD4 or CD8 form a heterogeneous CD3+/CD4–/CD8– T-cell population. One subset includes γδ T cells, which constitute 5%–9% of circulating CD3+ T cells in healthy adults (*10*,*11*). These percentages vary according to age and race/ethnicity (*12*) and have been reported to not exceed 5% of total peripheral CD3+ T cells in the population of the Czech Republic (*13*).

An increased proportion of peripheral blood γδ T cells was first reported in tularemia in 1992 (*14*). Subsequently, Kroca et al. observed that these T cells significantly increased in tularemia patients starting from the first week of symptom onset and persisted for months after resolution of illness (*15*). An increased frequency of γδ T cells has also been described in individual case reports or small case series in association with infections with other intracellular pathogens (*12*,*16*), including *Mycobacterium tuberculosis* (*17*), *Legionella* (*18*), *Salmonella* (*19*), *Brucella* (*20*,*21*), *Ehrlichia* (*22*), *Coxiella burnetii* (*23*), *Toxoplasma* (*24*), *Leishmania* (*25*), *Plasmodium vivax* (*26*), and *Schistosoma* spp. in primary schistosomiasis (*27*).

Encouraged by local colleagues suggesting that raised γδ T-cell (and by inference CD3+/CD4–/CD8– T-cell) percentages could be used in earlier tularemia diagnosis (*28*), the Immunology Laboratory of České Budějovice Hospital (České Budějovice, Czech Republic) changed its reporting practices in 2003. From this time onward, laboratory reports included comments suggesting consideration of possible tularemia if flow cytometry of peripheral blood showed an increased percentage of CD3+/CD4–/CD8– T cells or both CD3+/CD4–/CD8– and γδ T cells. In turn, clinicians in the 2 cooperating infectious disease units at České Budějovice Hospital and Písek Hospital (Písek, Czech Republic) began routinely requesting that peripheral blood samples be analyzed by flow cytometry along with the serologic test when tularemia was suspected.

In this article, we review cases under consideration for a tularemia diagnosis at these 2 infectious disease units to determine whether an increase in the percentage of CD3+/CD4–/CD8– T cells in the peripheral blood is sensitive and specific for a preliminary tularemia diagnosis and, if so, to define the optimum diagnostic cutoff value. Our second objective was to compare the timing of CD3+/CD4–/CD8– T-cell count elevation with that of the first positive *F*. *tularensis* serologic test result. We also evaluated the correlation between CD3+/CD4–/CD8– and γδ T cells to determine whether the levels of CD3+/CD4–/CD8– T cells could serve as a surrogate marker because this cell population is easier to assess.

## Methods

### Study Groups and Study Design

Using laboratory records and local hospital and unit diagnostic indices, we retrospectively identified all cases of tularemia that were managed in the infectious disease units at České Budějovice Hospital and Písek Hospital during January 1, 2003–December 31, 2015. The control group included a consecutive group of ill adults who were investigated for possible tularemia in the same 2 units during January 1, 2012–December 31, 2015.

We retrieved the hospital case notes for patients in each group. The study groups included patients for whom both tularemia serology and flow cytometry CD3+ T-cell population characterization were available during the same illness episode. We extracted data on demographics, signs and symptoms, final diagnoses, timing of symptom onset, and laboratory test results and recorded them onto a standardized form. Tularemia cases were categorized as probable or confirmed in keeping with published literature (*4*,*8*,*29*,*30*) and US Centers for Disease Control and Prevention 1999 and 2017 criteria (*31*,*32*): clinical illness compatible with tularemia along with detection of *F. tularensis* by culture or nucleic acid testing or a serologic test result suggestive of or confirming tularemia.

### Laboratory Diagnosis of Tularemia

For serologic testing, we used a commercial agglutination test (Tularemia Diagnostic Set, Bioveta a.s., https://www.bioveta.eu). We assigned a probable tularemia diagnosis to patients if the antibody titer in acute phase samples was >1:20 and illness was clinically compatible with tularemia. We assigned a confirmed tularemia diagnosis if the titer in any samples reached >1:160 or a seroconversion (change from negative to positive of any titer) or a 4-fold increase in titer occurred between the acute and convalescent samples and illness was clinically compatible with tularemia.

We performed blood cultures using BacT/ALERT 3D (bioMérieux, https://www.biomerieux.com); we cultured the resulting bacteria on plates with Columbia 5% sheep blood agar (Bio-Rad Laboratories, http://www.bio-rad.com) and determined the species by 16S PCR. For nucleic acid analysis, we extracted DNA using the QIAamp DNA Mini Kit (QIAGEN, https://www.qiagen.com) and used the panbacteria primers U3 and RU8 and thermocycler protocol for 16S PCR, in accordance with Radstrom et al. (*33*).

### Flow Cytometry

Staff of the Immunology Laboratory of České Budějovice Hospital, which acts as a reference laboratory for both participating infectious disease units, performed all tests. We stained EDTA-treated whole blood directly using CYTO-STAT tetraCHROME CD45-FITC (fluorescein isothiocyanate)/CD56-RD1 (phycoerythrin)/CD19-ECD (phycoerythrin-Texas Red-X)/CD3-PC5 (phycoerythrin cyanine 5), anti–CD4-Alexa Fluor 750, and anti–CD8-PC7 (phycoerythrin cyanine 7) (Beckman Coulter, https://www.beckmancoulter.com). For the subset of cases in which the CD3+/CD4–/CD8– T-cell percentage appeared high to the reporting bioscientist, the sample was further examined by staining with anti–CD3-FITC (fluorescein isothiocyanate) and anti–T-cell receptor PAN γδ-PE (phycoerythrin) (Beckman Coulter) on the same day.

**Figure 1:**
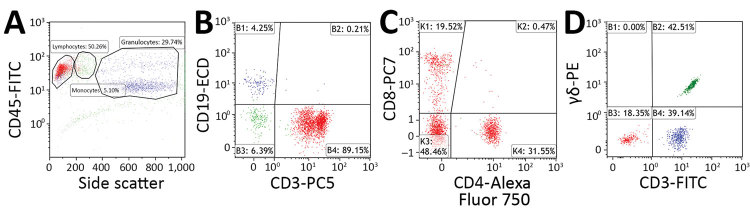
Flow cytometry gating strategy used to determine percentage of CD3+ lymphocytes that are CD3+/CD4–/CD8– T cells and γδ T cells in peripheral blood samples acquired from patients with suspected tularemia, Czech Republic, 2003–2015. A–C) Staining with CYTO-STAT tetraCHROME CD45-FITC/CD56-RD1 (phycoerythrin)/CD19-ECD/CD3-PC5, anti–CD4-Alexa Fluor 750, and anti–CD8-PC7 (Beckman Coulter, https://www.beckmancoulter.com). A) CD45 versus side scatter plot. Percentages of lymphocytes (red), monocytes (green), and granulocytes (blue) are indicated. In total, 3,000 lymphocytes were selected for further analysis. B) B cells (blue) and T cells (red) plotted according to their CD19 and CD3 expression. Percentages of cells within each quadrant are indicated. T cells were selected for further analysis. C) Percentage of CD3+ T cells not displaying CD4 and CD8 (CD4–/CD8–) determined with CD4 versus CD8 plots. Percentages of cells within each quadrant are indicated. D) Staining with anti–CD3-FITC and anti–T-cell receptor PAN γδ-PE (Beckman Coulter). After a side scatter and forward scatter plot (not shown), the percentage of lymphocytes that were CD3+/γδ T cells (green) were determined with a CD3 versus T-cell receptor pan–γδ plot. Percentages of cells within each quadrant are indicated. Flow cytometry was performed in the Immunology Laboratory of České Budějovice Hospital (České Budějovice, Czech Republic). ECD, phycoerythrin-Texas Red-X; FITC, fluorescein isothiocyanate; PC, phycoerythrin cyanine; PE, phycoerythrin.

We processed samples using either a Cytomics FC500 (before 2014) or Navios (starting in 2013) flow cytometer and CXP (for Cytomics FC500) or Navios software (Beckman Coulter for all). In the first gate, we selected 3,000 lymphocytes on the basis of their CD45 cell surface expression and side scatter characteristics (Figure 1, panel A). Next, we selected the T cells using a B- and T-cell plot gated according to CD19 and CD3 expression (Figure 1, panel B). Then, we identified the percentage of CD3+ T cells that did not express CD4 and CD8 in CD4 versus CD8 plots (Figure 1, panel C). When high percentages of CD3+/CD4–/CD8– T cells were found, we performed a subsequent staining and analysis using anti–CD3-FITC and anti–T-cell receptor PAN γδ-PE (Figure 1, panel D).

### Statistical Methods and Ethics Review

We tested the correlation between CD3+/CD4–/CD8– and γδ T cells in tularemia cases using the Spearman correlation coefficient. To examine cell count differences by diagnosis (probable vs. confirmed), we used the Mann-Whitney U test. To test differences among the different clinical manifestations, we used the Kruskal-Wallis test.

We compared the percentage of CD3+ lymphocytes with a CD4–/CD8– phenotype between the tularemia and control group patients. In the next analysis, we compared the timing of the first elevation of CD3+/CD4–/CD8– T cells with that of the first positive serologic test result for tularemia both relative to the reported day of symptom onset. We presented these results using medians and other nonparametric rank statistics. We evaluated the difference in percentage of CD3+/CD4–/CD8– T cells between tularemia cases and controls by the Mann-Whitney U test. To assess the predictive ability of flow cytometry, we generated a receiver operating characteristic (ROC) curve and calculated the area under the ROC curve. We determined the cutoff value yielding the highest sensitivity and specificity by using the Youden index (*34*).

We examined the difference in days between symptom onset to elevated CD3+/CD4–/CD8– percentage (using the cutoff value determined by the ROC curve) and symptom onset to first positive serologic test result by Wilcoxon signed rank test. We present all statistical parameters with their respective 95% CIs. We performed statistical analyses using IBM SPSS Statistics 24.0 (IBM Corp., https://www.ibm.com) and considered p values <0.05 significant. The post hoc test power was >80% in all tests.

The ethics committee at České Budějovice Hospital reviewed the study protocol and approved this study on December 20, 2013 (reference no. 17/2013). Consent of patients was not required for case note review.

## Results

### Tularemia Case Group

**Figure 2:**
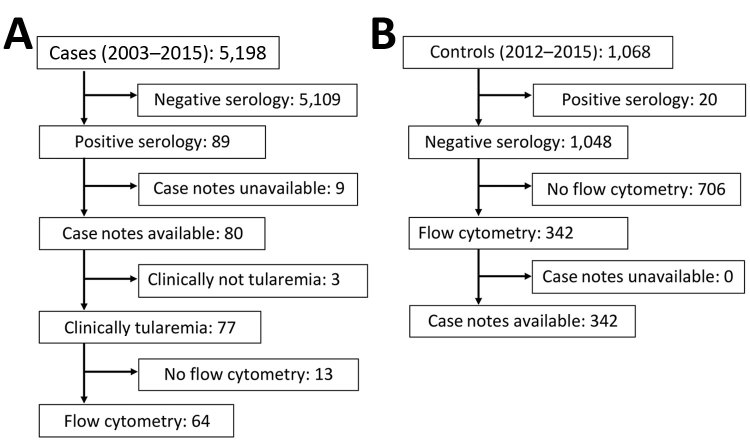
Selection of tularemia cases (2003–2015) and controls (2012–2015) in investigation of whether γδ T cells or CD3+/CD4–/CD8– T cells can be used for early presumptive tularemia diagnosis, Czech Republic.

During 2003–2015, we performed serologic tests for tularemia for 5,198 patients, and 89 patients had positive test results (Figure 2). After exclusion of 9 patients with missing case notes, 3 with resolved past infection, and 13 with missing flow cytometry results, 64 patients with tularemia had all the required information available for the same illness episode and could be included in the tularemia case group. Of 64 cases, 22 were defined as probable and 42 as confirmed (1 case positive by blood culture with a titer >1:160, 1 case positive by PCR with a seroconversion to titer <1:160, 13 cases of titer >1:160 including 7 seroconverters, and 27 cases of seroconversion to a titer <1:160) (Table 1).

**Table 1:** Clinical disease manifestation of tularemia patients, by type of diagnosis, Czech Republic, 2003–2015

Manifestation	No. probable cases, n = 22	No. confirmed cases, n = 42	No. (%) total cases, n = 64*
Ulceroglandular	7	22	29 (45.3)
Glandular	6	4	10 (15.6)
Oroglandular	2	7	9 (14.1)
Pulmonary	5	4	9 (14.1)
Typhoidal	2	5	7 (11.0)

*Percentages do not total 100% because of rounding.

Patients were treated according to local protocol with a drug combination of doxycycline and gentamicin or ciprofloxacin. Excision of a lymph node was necessary for 26.6% (17/64) of patients. All tularemia patients survived; median time to recovery was 50 (range 20–260) days. Five (7.8%) patients experienced a relapse and needed a second or prolonged course of antimicrobial drugs; 2 of these patients were treated with ciprofloxacin for <7 days and the remaining 3 with doxycycline and gentamicin for 3 weeks. The 3 patients needing prolonged drug treatment also required drainage of progressive purulent lymphadenopathy at the end of therapy.

### Control Group

During 2012–2015, tularemia serologic tests were performed for 1,068 consecutive adults; 20 of these patients had positive test results, 19 of whom had flow cytometry data available and were thus included in the tularemia case group. Of the remaining 1,048 patients with negative serologic test results, 342 had both case notes and flow cytometry results available. These patients, with various final diagnoses (Table 2), constituted the control group (Figure 2).

**Table 2:** Final diagnoses of 342 control group patients with negative serologic test results for tularemia and percentages of controls with elevated CD3+/CD4–/CD8– T cells, Czech Republic, 2012–2015*

Diagnosis	No. (%) controls†	No. (%) with elevated CD3+/CD4–/CD8– T cells
Nonspecific resolving lymphadenitis	99 (28.9)	7 (7.1)
Fever of unknown origin or fatigue	44 (12.9)	2 (4.5)
Epstein-Barr virus or cytomegalovirus	26 (7.6)	3 (11.5)
Lymphoma or cancer	26 (7.6)	1 (3.8)
*Chlamydia* or *Mycoplasma* respiratory infection	25 (7.3)	5 (20.0)
Recurring or nonresolving tonsilitis	17 (5.0)	4 (23.5)
Toxoplasmosis	16 (4.7)	5 (31.3)
Other‡	89 (26.0)	9 (10.1)§

*The percentage of the CD3+ T cells with a CD4–/CD8– phenotype was measured by flow cytometry and 8% was used as the cutoff value to define an elevated percentage. ANCA, anti-neutrophil cytoplasmic antibody. †Percentages do not total 100% because of rounding. ‡Other diagnoses: 8 cases each of cellulitis or skin abscess and lower respiratory tract infection or pneumonia; 5 cases of bartonellosis; 4 cases each of ANCA-positive vasculitis, leptospirosis, purulent sialadenitis, reactive arthritis (urethritis *C. trachomatis*), and tick-borne encephalitis; 3 cases of cervical cyst; 2 cases each of HIV, human granulocytic anaplasmosis, other reactive arthritis, polymyalgia rheumatica, sarcoidosis, systemic lupus erythematosus, toxocariasis, and viral meningitis; and 1 case each of acute sinusitis, ankylosing spondylitis, bacterial endocarditis, Behçet disease, cholangitis, deep vein thrombosis, dental abscess, erythema nodosum, farmer’s lung, hantavirus, herpetic tonsilitis, hidradenitis suppurativa, hyperthyroidism, hypothyroidism, legionellosis, lipoma, liver abscess, Lyme disease, mumps, necrotizing lymphadenitis of Kikuchi-Fujimoto, nonspecific hepatitis, parvovirus B19, pertussis, recurrent bacterial conjunctivitis, rickettsial disease, ulcerative colitis, undetermined tumor of the brain and pancreatic head, urinary tract infection, and *Yersinia enterocolitica* arthritis. §Includes 2 cases of arthralgia and 1 case each of ANCA-positive vasculitis, brain tumor, liver abscess, pneumocystis pneumonia in the setting of AIDS, rickettsial disease, skin and soft tissue infection, and toxocariasis.

Patients in the case group (median age 45 [range 16–66] years) were older than those in the control group (median age 36 [range 12–85] years; p<0.0001). More patients were male in the case group (67.2%, 43/64) than the control group (47.4%, 162/342; p = 0.004). All patients were white.

### Percentage of CD3+ T Cells with CD4*–/*CD8– Phenotype in Peripheral Blood Samples

**Figure 3:**
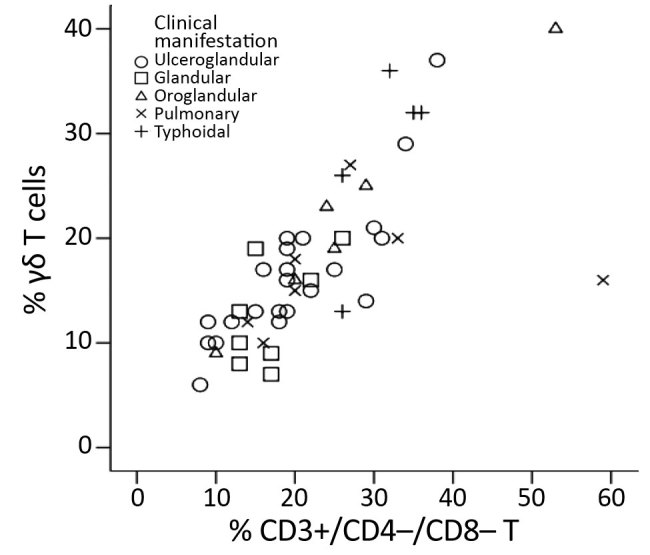
Correlation between percentage of CD3+ lymphocytes that are γδ T cells and percentage that are CD3+/CD4–/CD8– T cells in peripheral blood samples from patients with confirmed or probable tularemia diagnoses (n = 48), Czech Republic, 2003–2015. The Spearman correlation coefficient of this plot (0.830, 95% CI 0.679–0.906; p<0.0001) indicates a strong correlation and suggests that these T cells can be used interchangeably for tularemia diagnosis.

**Figure 4:**
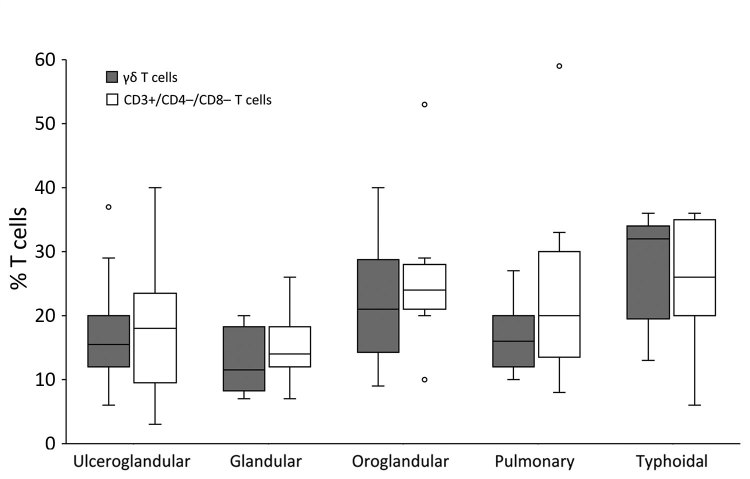
Percentage of CD3+ lymphocytes that are γδ T cells and CD3+/CD4–/CD8– T cells in peripheral blood samples from patients with confirmed or probable tularemia by clinical manifestation, Czech Republic, 2003–2015. The percentage of γδ T cells was determined for 48 cases and percentage of CD3+/CD4*–/*CD8– T cells for 64 cases. Paired comparisons (Kruskal-Wallis test) reveal no significant differences except for glandular versus typhoidal in γδ (p = 0.037) and CD3+/CD4*–*/CD8– T cells (p = 0.041). Boxes indicate interquartile ranges (IQRs), horizontal lines within boxes indicate medians, whiskers indicate range values <1.5× the IQR limits, and circles indicate outliers (i.e., values >1.5× the IQR limits).

The Spearman correlation coefficient of the plot of the CD3+/CD4*–/*CD8– T-cell and γδ T-cell percentages (in 48/64 tularemia cases where both parameters were known) was 0.830 (95% CI 0.679–0.906; p<0.0001) (Figure 3). This strong-positive correlation suggests these 2 measures can be used interchangeably. The percentages of these T cells did not differ by type of tularemia diagnosis (probable vs. confirmed; Mann-Whitney U test, p = 0.102 for CD3+/CD4*–/*CD8– and p = 0.364 for γδ). These subpopulations did differ by clinical manifestation (Kruskal-Wallis test, p = 0.041 for CD3+/CD4*–/*CD8– and p = 0.033 for γδ) (Figure 4), but the subgroups were too small for further analysis. Because of these results and the small sample size, we used the whole group of 64 probable and confirmed tularemia cases in further analyses.

**Figure 5:**
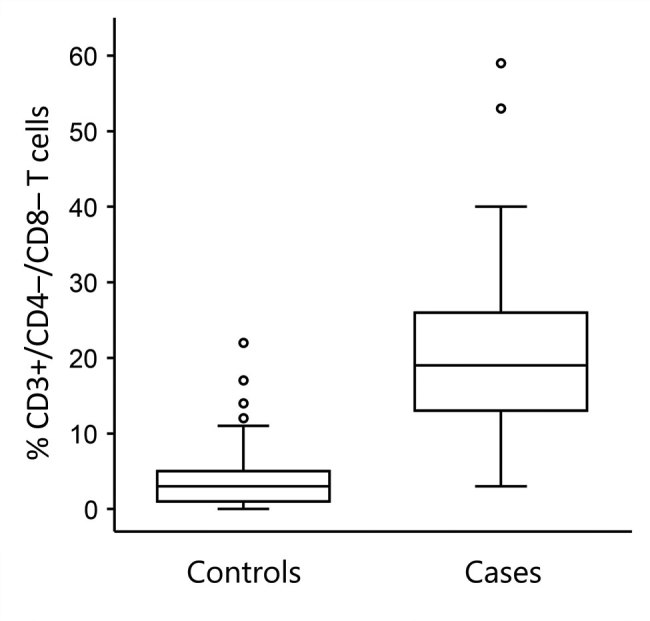
Comparison of percentages of CD3+ lymphocytes with CD4–/CD8– phenotype in peripheral blood samples from patients with probable or confirmed tularemia cases (n = 64, 2003–2015) and controls (n = 342, 2012–2015), Czech Republic. Boxes indicate interquartile ranges (IQRs), horizontal lines within boxes indicate medians, whiskers indicate range values <1.5× the IQR limits, and circles indicate outliers (i.e., values >1.5× times the IQR limits). The percentage of CD3+/CD4–/CD8– T cells is significantly higher in cases than controls (Mann-Whitney U test, p<0.0001).

**Figure 6:**
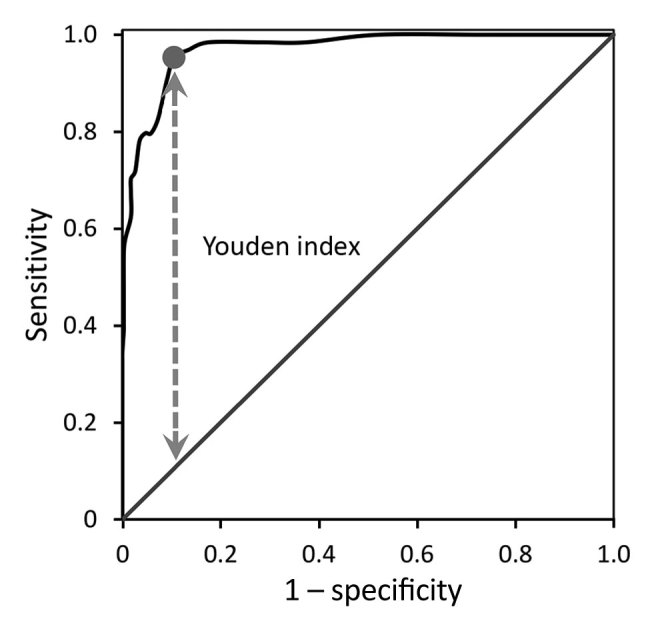
Receiver operating characteristic curve for diagnostic utility of raised CD3+/CD4–/CD8– T cells distinguishing probable and confirmed tularemia cases (n = 64, 2003–2015) from controls (n = 342, 2012–2015), Czech Republic. The area under the receiver operating characteristic curve is 0.970 (95% CI 0.952–0.988). The Youden index (circle on curve) is the maximal vertical distance (dashed line) of the curve from the diagonal line.

The percentage of CD3+/CD4–/CD8– T cells was higher in the case group (median 19%, 95% CI 17%–22%, interquartile range 13%–26%) than the control group (median 3%, 95% CI 2%–3%, interquartile range 1%–5%; Mann-Whitney U = 652.5, *Z* = –12.02, p<0.0001) (Figure 5). The area under the ROC curve assessing the sensitivity and specificity of this flow cytometry–based diagnostic test was 0.970 (95% CI 0.952–0.988). The optimum cutoff CD3+/CD4–/CD8– T-cell percentage was 8%, and with this cutoff, the sensitivity was 95.3% (95% CI 88.0%–98.7%) and specificity was 89.5% (95% CI 85.9%–92.4%) for discriminating between cases and controls (Figure 6). Among our cohort, 61 (95.3%) tularemia patients and 36 (10.5%) controls had a peripheral blood CD3+/CD4–/CD8– T-cell percentage >8% (Table 2).

### Timing in Rise of CD3+/CD4*–*/CD8– T-Cell Percentage and First Positive Serologic Test Result

**Figure 7:**
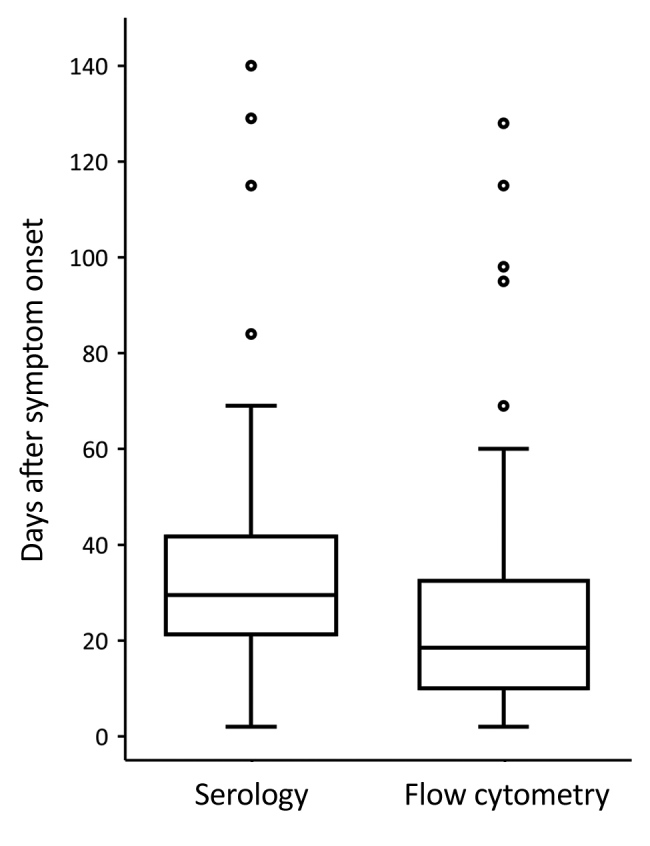
Comparison of time to first positive serologic test result for tularemia and time to raised CD3+/CD4–/CD8– T-cell percentage determined by flow cytometry relative to the time of symptom onset of 58 patients with probable or confirmed tularemia, Czech Republic, 2003–2015. Percentages of CD3+/CD4–/CD8– T cells >8% were considered raised. A positive serologic test result for tularemia was defined for probable cases as an antibody titer of >1:20 in any acute phase blood sample and for confirmed cases as a single antibody titer of >1:160 in any blood sample or a seroconversion from negative to positive (any titer) or a 4-fold increase in titer between acute and convalescent patient samples (agglutination test; Tularemia Diagnostic Set, Bioveta a.s., https://www.bioveta.eu). Boxes indicate interquartile ranges (IQRs), horizontal lines within boxes indicate medians, whiskers indicate range values <1.5× the IQR limits, and circles indicate outliers (i.e., values >1.5× times the IQR limits). The CD3+/CD4–/CD8– T cells increased before *Francisella tularensis*–specific antibody titers increased (Wilcoxon signed rank test, p<0.0001).

The time of symptom onset was known for 58 of 61 tularemia patients with CD3+/CD4–/CD8– T-cell percentages >8%. Among these 58 patients, the increased CD3+/CD4–/CD8– T-cell percentage preceded the first positive serologic test result by a median of 7 (95% CI 1.0–12.0) days (Wilcoxon signed rank test *Z* = –4.796; p<0.0001) (Table 3; Figure 7). In the subset of tularemia patients in whom an elevated CD3+/CD4–/CD8– T-cell percentage was detected while serology was still negative (58.6%, 34/58), the median delay from rise of CD3+/CD4–/CD8– T cells to positive serologic test result was 14 (95% CI 8–22) days (Table 3).

**Table 3:** Time to positive diagnostic test result for tularemia, by starting time point, test, and population, Czech Republic, 2003–2015*

Category	Time, d			
	Median	95% CI	Interquartile range	Range
Time relative to onset of patient symptoms				
Diagnostic test type				
Flow cytometry, n = 58	18.5	15.5–22.0	9.75–33.25	2–128
Serologic test, n = 58	29.5	24.0–37.0	21.0–42.0	2–140
Time to first positive serologic test result relative to rise in CD3+/CD4–/CD8– T cells				
Patient population				
All, n = 58	7.0	1.0–12.0	0–18.75	–50 to 62
Delayed seroconverters, n = 34	14.0	8.0–22.0	7.5–22.0	1–62

*A positive flow cytometry test result for tularemia was defined as >8% of peripheral blood CD3+ T cells having the CD4–/CD8– phenotype. A positive serologic test result for tularemia included probable and confirmed diagnoses and was defined for probable cases as an antibody titer of >1:20 in any acute phase blood sample or for confirmed cases as an antibody titer of >1:160 in any blood sample or a seroconversion from negative to positive (any titer) or a 4-fold increase in titer between acute and convalescent patient samples (agglutination test; Tularemia Diagnostic Set, Bioveta a.s., https://www.bioveta.eu).

### Comparison of Cases and Controls in 2012–2015 Only

To investigate the possible effects of bias introduced by comparing cases selected from a 13-year period with controls from just the last 4 years of that period, we repeated our analyses with the 19 tularemia patients and 342 controls with full data available who sought treatment during 2012–2015. When we used 8% as the cutoff, we found the percentage of CD3+/CD4–/CD8– T cells was raised in 100% (19/19) of cases and 10.5% (36/342) of controls. The 8% cutoff value had a sensitivity of 100% (95% CI 87.8%–100%) and specificity of 89.5% (95% CI 85.9%–92.4%) for distinguishing tularemia patients from controls seeking treatment during this period.

## Discussion

This study confirms results of earlier reports (*14*,*15*,*35*) describing the potential application of flow cytometry to support early presumptive tularemia diagnosis. We showed that CD3+/CD4–/CD8– T cells can be used as a pragmatic surrogate for γδ T cells in this context. The percentage of CD3+ T cells with the CD4–/CD8– phenotype was 19% in tularemia patients with differing disease presentations and 3% in control patients with a wide variety of infectious and noninfectious conditions. When we used 8% as the cutoff to define elevated CD3+/CD4–/CD8– T cells, we found this flow cytometry–based test was elevated >7 days before serologic test results became positive and had a sensitivity of 95% and specificity of 89.5% for distinguishing tularemia cases from other illnesses.

Flow cytometry is not routinely used to investigate most infections but is available in centers that care for HIV patients. The CD3+/CD4–/CD8– T-cell percentage is easily measurable and can be performed before patients seroconvert or while awaiting serologic test results (*30*,*36*). One of the strengths of this study was that the control group consisted of patients with a wide variety of illnesses, rather than a group of healthy volunteers, as in previous reports. These patients were investigated for possible tularemia as part of routine care over several years, representing real-life practice.

Our study was conducted in the Czech Republic, a setting with a low incidence of diseases that might be mistaken for tularemia, such as brucellosis, leptospirosis, Q fever, tuberculosis, or malaria (*12*); our study conclusions are probably most applicable to settings with low prevalences of the intracellular pathogens that cause these diseases. This study should be repeated in other geographic areas with higher prevalences of these infections (*21*), which could decrease the sensitivity and specificity of this test for predicting tularemia diagnoses.

Test results might need to be considered with more caution in less racially homogeneous populations because the reference range of CD3+/CD4–/CD8– T cells can vary by race/ethnicity and age (*12*). A study conducted in the United States showed a higher baseline percentage of γδ T cells in healthy white persons (3.7%) than in healthy black persons (1.18%) (*11*), and a study in Sweden indicated a lower percentage in persons from Sweden (4.2%) and Japan (4.5%) than from Bangladesh (9.2%) or Turkey (9.3%) (*10*). Higher percentages of γδ T cells in inhabitants of West Africa might be a result of priming during childhood with malaria parasites (*37*). Likewise, a higher percentage of baseline γδ T cells in populations of Turkey might be caused by Turkey’s higher prevalence of latent tuberculosis compared with central Europe (*10*). The CD3+/CD4–/CD8– T-cell percentage might be valuable to use as a generic marker for intracellular infections when investigating fever of unknown origin, although specificity might not be adequate because these percentages can also be elevated in association with some cancers (*12*).

Because of the relative rarity of tularemia, we used a retrospective design to enroll sufficient numbers of tularemia patients into this pilot study, and adequate details were not available for 26% (22/86) of patients. However, the spectrum of clinical presentations of those included is representative of tularemia patients in other published series in the Czech Republic (*38*) and elsewhere (*4*,*7*), and bias seems unlikely. The mix of final diagnoses in the control group gives reasonable confidence that other intracellular infections, cancers, and noninfectious causes of fever and lymphadenopathy did not substantially contribute to false-positive early presumptive tularemia diagnoses.

We used the data of tularemia patients treated during 2003–2015 and control patients treated during 2012–2015. The use of patients from different periods might have introduced selection bias, but this bias should have been mitigated by the large number of control patients included with complete data available and by the variety of control patient illnesses. That this bias was minimal is supported by the reproducibility of our findings when we performed a comparative analysis restricted to just the tularemia patients and controls treated during 2012–2015.

Serology has been and remains the standard test for tularemia diagnosis for many disease presentations. Other diagnostic tests have drawbacks. The organism is difficult to culture, as demonstrated by only 1 case in this series having a positive blood culture result. Although PCR with an ulcer swab sample resulted in DNA amplification in 1 case, PCR amplification failed in other cases. Also, only 45.4% of our patient cohort had an easily accessible lesion to swab. We believe that flow cytometry can contribute to tularemia diagnosis better than these other available tests with minimal invasiveness and cost, even when PCR methods become more widely available. In addition, the expansion in the use of PCR will not result in faster tularemia diagnosis because the clinician still needs to suspect tularemia before requesting this test be performed. However, PCR could be used as a confirmatory test for cases with elevated percentages of CD3+/CD4–/CD8– T cells.

The retrospective nature of this study limited our investigations regarding the timing of CD3+/CD4–/CD8– T-cell elevations and *F. tularensis* antibody titer increases in relation to the onset of patient symptoms. In all cases, the flow cytometry test result recorded was that from the first flow cytometry test performed. In contrast, for tularemia serology, the first positive serologic test result was recorded. Most tests were requested when the differential diagnosis first included tularemia. In a small proportion of patients, flow cytometry was performed after a positive serologic test result for tularemia was communicated to the physician; thus, for these patients, flow cytometry results were delayed. The time from symptom onset to elevation of CD3+/CD4–/CD8– T cells that we report does not reflect the timing this cell population increases and how soon this flow cytometry–based diagnostic test can be performed. In addition, in some cases, the diagnostic work-up for tularemia was delayed because of delayed referral of patients to the infectious diseases unit of the hospital. Therefore, we cannot comment on the reliability of the flow cytometry–based method during the first week after symptom onset.

In our study, the rise in the percentage of CD3+/CD4–/CD8– T cells preceded seroconversion even in patients with late referrals. Seroconversion was documented in 53.1% (34/64) of patients with tularemia, many of whom had been treated for tularemia on the basis of raised CD3+/CD4*–/*CD8– T-cell percentages and were monitored until seroconversion or an alternative diagnosis was obtained. Including flow cytometry in the tularemia work-up for our cohort contributed to the high percentage of diagnoses confirmed by seroconversion (53.1%), which was much higher than those of other cohorts: 0% in Missouri, USA (*7*), and Turkey (*30*); 5% (5/101) in France (*8*); 35% (9/26) in Sweden (*39*); and 13.4% (19/142) in Spain (*40*).

In 25% of the tularemia cases (Table 3), the time from symptom onset to first positive serologic test result was >22 days, occurring >14 days after the detection of an elevated CD3+/CD4–/CD8– T-cell percentage, which lends strong support to the use of flow cytometry to identify suspected cases for empirical treatment. Because tularemia might not have pathognomonic manifestations, adding an extra tube for flow cytometry as part of the diagnostic work-up can help physicians make decisions regarding whether to treat a given health condition as tularemia in cases where serologic results are still negative and the team is waiting for PCR or blood culture results.

In conclusion, in the Czech Republic, flow cytometry analyses of peripheral blood samples showing a percentage of CD3+/CD4–/CD8– T cells >8% supports a presumptive clinical diagnosis of tularemia and initiation of specific antimicrobial therapy days to weeks before the diagnosis can be confirmed serologically. This more rapid test is a useful addition to the diagnostic work-up for tularemia that can help public health teams managing waterborne outbreaks and inhalation infection clusters speed up diagnosis and treatment and thus contain pathogen spread. In hospital settings, the rapid diagnosis of tularemia afforded with this test might indicate the need for Biosafety Level 3 facilities, required for *F. tularensis* propagation, thereby reducing occupational health risk.

## References

[1] McCoy GW, Chapin CW. Further observations on a plague-like disease of rodents with a preliminary note on the causative agent, *Bacterium tularense.* J Infect Dis. 1912;10:61–72. 10.1093/infdis/10.1.61

[2] European Centre for Disease Prevention and Control. Tularaemia-annual epidemiological report 2016. 2019 Jan 22 [cited 2018 Dec 7]. https://ecdc.europa.eu/sites/portal/files/documents/AER_for_2016-tularaemia.pdf

[3] Sjöstedt A. Tularemia: history, epidemiology, pathogen physiology, and clinical manifestations. Ann N Y Acad Sci. 2007;1105:1–29. 10.1196/annals.1409.009 17395726

[4] Maurin M, Gyuranecz M. Tularaemia: clinical aspects in Europe. Lancet Infect Dis. 2016;16:113–24. 10.1016/S1473-3099(15)00355-2 26738841

[5] Yapar D, Erenler AK, Terzi Ö, Akdoğan Ö, Ece Y, Baykam N. Predicting tularemia with clinical, laboratory and demographical findings in the ED. Am J Emerg Med. 2016;34:218–21. 10.1016/j.ajem.2015.10.034 26577431

[6] Tuncer E, Onal B, Simsek G, Elagoz S, Sahpaz A, Kilic S, et al. Tularemia: potential role of cytopathology in differential diagnosis of cervical lymphadenitis: multicenter experience in 53 cases and literature review. APMIS. 2014;122:236–42. 10.1111/apm.12132 23763361

[7] Weber IB, Turabelidze G, Patrick S, Griffith KS, Kugeler KJ, Mead PS. Clinical recognition and management of tularemia in Missouri: a retrospective records review of 121 cases. Clin Infect Dis. 2012;55:1283–90. 10.1093/cid/cis706 22911645

[8] Maurin M, Pelloux I, Brion JP, Del Banõ JN, Picard A. Human tularemia in France, 2006-2010. Clin Infect Dis. 2011;53:e133–41. 10.1093/cid/cir612 22002987

[9] Chu M, Elkins K, Nano F, Titball R. Considerations for handling *F. tularensis*. In: Tärnvik A, editor. WHO guidelines on tularaemia. Geneva: World Health Organization; 2007 [cited 2018 Dec 7]. https://www.who.int/csr/resources/publications/WHO_CDS_EPR_2007_7.pdf?ua=1

[10] Esin S, Shigematsu M, Nagai S, Eklund A, Wigzell H, Grunewald J. Different percentages of peripheral blood γ δ ^+^ T cells in healthy individuals from different areas of the world. Scand J Immunol. 1996;43:593–6. 10.1046/j.1365-3083.1996.d01-79.x 8633219

[11] Cairo C, Armstrong CL, Cummings JS, Deetz CO, Tan M, Lu C, et al. Impact of age, gender, and race on circulating γδ T cells. Hum Immunol. 2010;71:968–75. 10.1016/j.humimm.2010.06.014 20600446 PMC2941533

[12] Bank I, Marcu-Malina V. Quantitative peripheral blood perturbations of γδ T cells in human disease and their clinical implications. Clin Rev Allergy Immunol. 2014;47:311–33. 10.1007/s12016-013-8391-x 24126758

[13] Cibulka M, Selingerová I, Fědorová L, Zdražilová Dubská L. Immunological aspects in oncology–circulating γδ T-cells [in Czech]. Klin Onkol. 2015;28(Suppl 2):S60–8. 10.14735/amko20152S60 26374160

[14] Sumida T, Maeda T, Takahashi H, Yoshida S, Yonaha F, Sakamoto A, et al. Predominant expansion of V γ 9/V δ 2 T cells in a tularemia patient. Infect Immun. 1992;60:2554–8. 1534075 10.1128/iai.60.6.2554-2558.1992 PMC257198

[15] Kroca M, Tärnvik A, Sjöstedt A. The proportion of circulating gammadelta T cells increases after the first week of onset of tularaemia and remains elevated for more than a year. Clin Exp Immunol. 2000;120:280–4. 10.1046/j.1365-2249.2000.01215.x 10792377 PMC1905656

[16] Chen ZW, Letvin NL. Vgamma2Vdelta2+ T cells and anti-microbial immune responses. Microbes Infect. 2003;5:491–8. 10.1016/S1286-4579(03)00074-1 12758278 PMC2873077

[17] Tsukaguchi K, Balaji KN, Boom WH. CD4+ alpha beta T cell and gamma delta T cell responses to *Mycobacterium tuberculosis*. Similarities and differences in Ag recognition, cytotoxic effector function, and cytokine production. J Immunol. 1995;154:1786–96. 7836763

[18] Kroca M, Johansson A, Sjöstedt A, Tärnvik A. V γ 9V δ 2 T cells in human legionellosis. Clin Diagn Lab Immunol. 2001;8:949–54. 11527809 10.1128/CDLI.8.5.949-954.2001 PMC96177

[19] Hara T, Mizuno Y, Takaki K, Takada H, Akeda H, Aoki T, et al. Predominant activation and expansion of V gamma 9-bearing gamma delta T cells in vivo as well as in vitro in *Salmonella* infection. J Clin Invest. 1992;90:204–10. 10.1172/JCI115837 1386086 PMC443082

[20] Bertotto A, Gerli R, Spinozzi F, Muscat C, Scalise F, Castellucci G, et al. Lymphocytes bearing the γ δ T cell receptor in acute *Brucella melitensis* infection. Eur J Immunol. 1993;23:1177–80. 10.1002/eji.1830230531 8477812

[21] Kilic SS, Akbulut HH, Ozden M, Bulut V. Gamma/delta T cells in patients with acute brucellosis. Clin Exp Med. 2009;9:101–4. 10.1007/s10238-008-0021-1 19048184

[22] Caldwell CW, Everett ED, McDonald G, Yesus YW, Roland WE. Lymphocytosis of γ/δ T cells in human ehrlichiosis. Am J Clin Pathol. 1995;103:761–6. 10.1093/ajcp/103.6.761 7785663

[23] Schneider T, Jahn HU, Liesenfeld O, Steinhoff D, Riecken EO, Zeitz M, et al. The number and proportion of Vgamma9 Vdelta2 T cells rise significantly in the peripheral blood of patients after the onset of acute Coxiella burnetii infection. Clin Infect Dis. 1997;24:261–4. 10.1093/clinids/24.2.261 9114159

[24] Scalise F, Gerli R, Castellucci G, Spinozzi F, Fabietti GM, Crupi S, et al. Lymphocytes bearing the gamma delta T-cell receptor in acute toxoplasmosis. Immunology. 1992;76:668–70. 1398756 PMC1421574

[25] Russo DM, Armitage RJ, Barral-Netto M, Barral A, Grabstein KH, Reed SG. Antigen-reactive gamma delta T cells in human leishmaniasis. J Immunol. 1993;151:3712–8. 8376802

[26] Perera MK, Carter R, Goonewardene R, Mendis KN. Transient increase in circulating gamma/delta T cells during *Plasmodium vivax* malarial paroxysms. J Exp Med. 1994;179:311–5. 10.1084/jem.179.1.311 8270875 PMC2191326

[27] Schwartz E, Rosenthal E, Bank I. Gamma delta T cells in non-immune patients during primary schistosomal infection. Immun Inflamm Dis. 2014;2:56–61. 10.1002/iid3.18 25400925 PMC4220667

[28] Bártová V, Žampach P. Certain immune parameters in tularemia [in Czech]. Klin Mikrobiol Infekc Lek. 2000;6: 77–8.

[29] Mailles A, Vaillant V. 10 years of surveillance of human tularaemia in France. Euro Surveill. 2014;19:20956. 10.2807/1560-7917.ES2014.19.45.20956 25411688

[30] Erdem H, Ozturk-Engin D, Yesilyurt M, Karabay O, Elaldi N, Celebi G, et al. Evaluation of tularaemia courses: a multicentre study from Turkey. Clin Microbiol Infect. 2014;20:O1042–51. 10.1111/1469-0691.12741 24975504

[31] Centers for Disease Control and Prevention. Tularemia (*Francisella tularensis*). 1999 case definition. [cited 2018 Aug 29]. https://wwwn.cdc.gov/nndss/conditions/tularemia/case-definition/1999

[32] Centers for Disease Control and Prevention. Tularemia (*Francisella tularensis*). 2017 case definition. [cited 2018 Aug 29]. https://wwwn.cdc.gov/nndss/conditions/tularemia/case-definition/2017

[33] Rådström P, Bäckman A, Qian N, Kragsbjerg P, Påhlson C, Olcén P. Detection of bacterial DNA in cerebrospinal fluid by an assay for simultaneous detection of *Neisseria meningitidis*, *Haemophilus influenzae*, and streptococci using a seminested PCR strategy. J Clin Microbiol. 1994;32:2738–44. 7852565 10.1128/jcm.32.11.2738-2744.1994 PMC264152

[34] Youden WJ. Index for rating diagnostic tests. Cancer. 1950;3:32–5. 10.1002/1097-0142(1950)3:1<32::AID-CNCR2820030106>3.0.CO;2-3 15405679

[35] Poquet Y, Kroca M, Halary F, Stenmark S, Peyrat MA, Bonneville M, et al. Expansion of Vgamma9 Vdelta2 T cells is triggered by Francisella tularensis-derived phosphoantigens in tularemia but not after tularemia vaccination. Infect Immun. 1998;66:2107–14. 9573096 10.1128/iai.66.5.2107-2114.1998 PMC108170

[36] Bulut OC, Dyckhoff G, Splettstoesser W, Nemeth J, Klauschen F, Penzel R, et al. Unmasked: when a clinically malignant disease turns out infectious. A rare case of tularemia. Int J Surg Pathol. 2013;21:76–81. 10.1177/1066896912448424 22674915

[37] Hviid L, Akanmori BD, Loizon S, Kurtzhals JA, Ricke CH, Lim A, et al. High frequency of circulating γ δ T cells with dominance of the v(_δ)_1 subset in a healthy population. Int Immunol. 2000;12:797–805. 10.1093/intimm/12.6.797 10837407

[38] Cerný Z. Changes of the epidemiology and the clinical picture of tularemia in Southern Moravia (the Czech Republic) during the period 1936-1999. Eur J Epidemiol. 2001;17:637–42. 10.1023/A:1015551213151 12086077

[39] Strålin K, Eliasson H, Bäck E. An outbreak of primary pneumonic tularemia. N Engl J Med. 2002;346:1027–9, author reply 1027–9. 10.1056/NEJM200203283461316 11919317

[40] Pérez-Castrillón JL, Bachiller-Luque P, Martín-Luquero M, Mena-Martín FJ, Herreros V. Tularemia epidemic in northwestern Spain: clinical description and therapeutic response. Clin Infect Dis. 2001;33:573–6. 10.1086/322601 11462198

